# Nemaline Myopathy-Related Skeletal Muscle α-Actin (*ACTA1*) Mutation, Asp286Gly, Prevents Proper Strong Myosin Binding and Triggers Muscle Weakness

**DOI:** 10.1371/journal.pone.0045923

**Published:** 2012-09-20

**Authors:** Julien Ochala, Gianina Ravenscroft, Nigel G. Laing, Kristen J. Nowak

**Affiliations:** 1 Department of Neuroscience, Clinical Neurophysiology, Uppsala University, Uppsala, Sweden; 2 Centre for Medical Research, The University of Western Australia, Western Australian Institute for Medical Research, Nedlands, Australia; Semmelweis University, Hungary

## Abstract

Many mutations in the skeletal muscle α-actin gene (*ACTA1*) lead to muscle weakness and nemaline myopathy. Despite increasing clinical and scientific interest, the molecular and cellular pathogenesis of weakness remains unclear. Therefore, in the present study, we aimed at unraveling these mechanisms using muscles from a transgenic mouse model of nemaline myopathy expressing the *ACTA1* Asp286Gly mutation. We recorded and analyzed the mechanics of membrane-permeabilized single muscle fibers. We also performed molecular energy state computations in the presence or absence of Asp286Gly. Results demonstrated that during contraction, the Asp286Gly acts as a “poison-protein” and according to the computational analysis it modifies the actin-actin interface. This phenomenon is likely to prevent proper myosin cross-bridge binding, limiting the fraction of actomyosin interactions in the strong binding state. At the cell level, this decreases the force-generating capacity, and, overall, induces muscle weakness. To counterbalance such negative events, future potential therapeutic strategies may focus on the inappropriate actin-actin interface or myosin binding.

## Introduction

Nemaline myopathy (NM) is an under-appreciated congenital muscular disorder characterized clinically by skeletal muscle weakness and hypotonia; and pathologically by the presence of nemaline bodies within the muscle biopsy specimens [1]. NM has been shown to be caused by mutations in multiple different genes, including *ACTA1* (encoding skeletal muscle α-actin) [2], *NEB* (nebulin) [3], *TPM2* (β-tropomyosin) [4], *TPM3* (α-tropomyosin) [5], *TNNT1* (slow skeletal muscle troponin T) [6], *CFL2* (muscle specific cofilin) [7] and *KBTBD13* [8]. Despite this crucial knowledge, it remains unclear how most mutations disrupt muscle contractile function and lead to skeletal muscle weakness. Such information is of primary importance in order to design potential therapeutic interventions.

We recently developed a transgenic mouse model that mimics NM caused by *ACTA1* mutations [9]. The Tg(*ACTA1*)^Asp286Gly^ mice carry a missense *ACTA1* mutation that results in the substitution of one single amino acid in the actin protein (Asp286Gly in the mature protein, Asp288Gly according to the standard Human Genome Variation Society nomenclature) that was identified in a NM patient [9]. Tg(*ACTA1*)^Asp286Gly^ mice are weaker than wild-type mice and their skeletal muscles display numerous pathological lesions characteristic of human patients diagnosed with NM [9]. The severely depressed steady-state isometric force at the muscle fiber level and the overall skeletal weakness in these animals have been hypothesized to be due both to effects of (i) the mutant protein interfering directly “with the ability of the sarcomeres to produce force” and (ii) the presence of non-contractile areas (nemaline bodies) decreasing the number of functional sarcomeres [9], [10]. To examine the accuracy of these two hypotheses, in the present study, we aimed at characterizing Asp286Gly-associated functional consequences by recording and analyzing the mechanics of single membrane-permeabilized muscle fibers from wild-type and Tg(*ACTA1*)^Asp286Gly^ mice. In addition, to gain molecular insights into the dysfunction, we performed molecular energy computations in the presence or absence of Asp286Gly.

## Materials and Methods

### Animals

Skeletal muscles (extensor digitorum longus, EDL) were dissected from three to four-month old male wild-type and age-matched Tg(*ACTA1*)^Asp286Gly^ mice after sacrifice. For a complete description of the transgenic mice expressing the *ACTA1* Asp286Gly defect, please refer to [9]. All procedures involving animal care, welfare and handling were performed according to institutional guidelines and were reviewed and approved by the Animal Ethics Committee of The University of Western Australia (RA03/100/918).

**Table 1:** Solutions used for the mechanical recordings (ionic strength: 180 mM, pH: 7.0).

	pCa	KCl	Imidazole	MgCl_2_	EGTA	CaCl_2_	CrP	ATP
**Relaxing solution**	9.0	77.63	20.00	5.46	7.00	17.70.10^−3^	14.50	4.65
**Pre-activating solution**	9.0	77.63	20.00	5.46	0.50	17.70.10^−3^	14.50	4.65
**Activating solutions**	Up to 4.5	62.70	20.00	5.27	7.00	Up to 7.00	14.50	4.71
**Rigor solution**	4.5	62.70	20.00	5.27	7.00	7.00	-	-

Concentrations are presented in mM.

**Figure 1:**
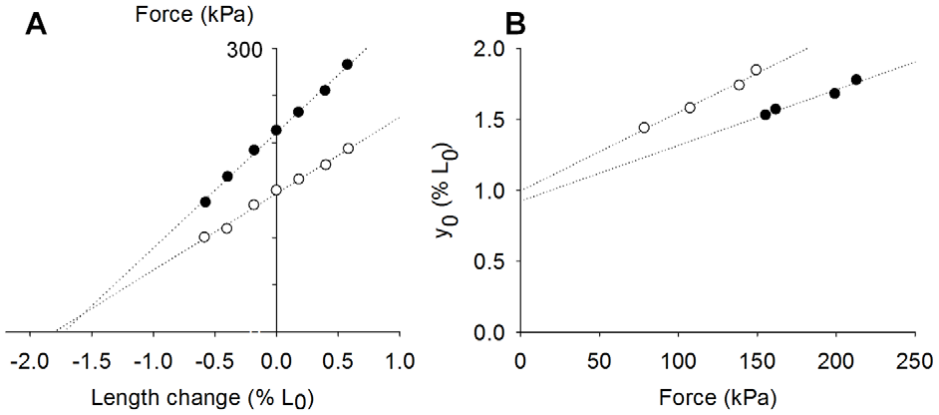
Typical stiffness data. [A] represents the peak force-length change relationships as well as the resultant y_0_-force curve of fibers from a wild-type animal (•) and from a Tg(*ACTA1*)^Asp286Gly^ mouse (○).

### Skeletal muscle sample preparation

Skeletal muscle samples were placed in relaxing solution at 4°C. Bundles of approximately 50 fibers were dissected free and then tied with surgical silk to glass capillary tubes at slightly stretched lengths. They were then treated with skinning solution (relaxing solution containing glycerol; 50∶50 v/v) for 24 hours at 4°C, after which they were transferred to −20°C. In addition, the muscle bundles were treated with sucrose, a cryoprotectant, within 1–2 weeks for long-term storage [11]. They were detached from the capillary tubes and snap frozen in liquid nitrogen-chilled propane and stored at −160°C.

**Table 2:** Mechanical parameters of fibers from Tg(*ACTA1*)^Asp286Gly^ and wild-type mice during steady-state isometric contractions.

	Tg(*ACTA1*)^Asp286Gly^	Wild-type
**Maximal force (kPa)**	124.20±12.40^#^ (n = 34)	221.80±10.70 (n = 35)
**Active stiffness (kPa.% L_0_^−1^)**	51.60±5.70^#^ (n = 22)	112.10±15.70 (n = 24)
**Rigor stiffness (kPa.% L_0_^−1^)**	161.20±16.10 (n = 22)	187.40±14.50 (n = 24)
***f*** **_xb_ (A.U.)**	0.24±0.03^#^ (n = 22)	0.42±0.04 (n = 24)
**y_0_** * ** (% L_0_)**	1.01±0.07 (n = 20)	1.07±0.23 (n = 20)
**C_f_+C_e_ (% L_0_.MPa^−1^)**	6.50±1.30 (n = 20)	5.30±0.90 (n = 20)
***k*** **_tr_ (s^−1^)**	52.90±5.30 (n = 12)	41.70±2.90 (n = 11)
**V_0_ (ML.s^−1^)**	5.80±0.70^#^ (n = 10)	3.90±0.80 (n = 10)

Due to a type IIb muscle fiber predominance in the EDL muscles, the comparison was restricted to cells expressing the type IIb MyHC isoform. ^#^ indicates a significant difference when compared with wild-type animals (p<0.05). For each parameter, n represents the number of tested fibers.

**Figure 2:**
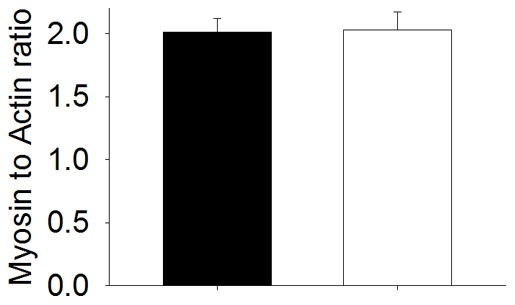
Actomyosin content in presence of Asp286Gly. It depicts the myosin to actin ratio. Values are presented as means (bars are standard errors) and ▪ correspond to data from from wild-type mice whereas □ are from Tg(*ACTA1*)^Asp286Gly^ mice.

### Mechanical recordings and analyses

On the day of experimentation, bundles were de-sucrosed, transferred to a relaxing solution and single fibers dissected. A fiber 1 to 2 mm long was placed between connectors leading to a force transducer (model 400A, Aurora Scientific) and a lever arm system (model 308B, Aurora Scientific) [12], [13]. The two extremities of the fiber were tightly attached to the connectors as previously described [12]. The apparatus was mounted on the stage of an inverted microscope (model IX70; Olympus). The sarcomere length was set to 2.50–2.60 µm (optimal mouse sarcomere length where the force production is the highest) and controlled during the experiment using a high-speed video analysis system (model 901A HVSL, Aurora Scientific). The diameter of the fiber between the connectors was measured through the microscope at a magnification of ×320 with an image analysis system prior to the mechanical experiments. Fiber depth was measured by recording the vertical displacement of the microscope nosepiece while focusing on the top and bottom surfaces of the fiber. The focusing control of the microscope was used as a micrometer. Cross-sectional area (CSA) was calculated from the diameter and depth, assuming an elliptical circumference, and was corrected for the 20% swelling that is known to occur during skinning [12]. Diameter and depth were measured at three different locations along the length of each fiber and the mean was considered as representative of cell dimensions. As previously described in details [14], [15], [16], mechanical experiments were done at 15°C and included force measurements (adjusted to CSA) after various length steps. All the solutions used are presented in Table 1.

**Figure 3:**
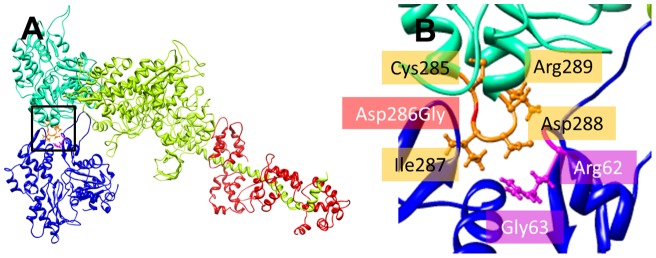
Actomyosin complex in presence of Asp286Gly. General [A] and particular view [B] of the actin residues with modified energies (non-bonded and electrostatic interactions). The actomyosin model used here was originally designed by Lorenz and Holmes [17].

### Protein expression and quantification

After mechanical recordings, each fiber was placed in urea buffer (120 g urea, 38 g thiourea, 70 ml H_2_0, 25 g mixed bed resin, 2.89 g dithiothreitol, 1.51 g Trizma base, 7.5 g SDS, 0.004 % bromophenol blue) in a plastic microcentrifuge tube and stored at −160°C. Myosin heavy chain (MyHC) isoform composition of fibers was then determined by 6% SDS-PAGE. The acrylamide concentration was 4% (wt/vol) in the stacking gel and 6% in the running gel, and the gel matrix included 30% glycerol. Sample loads were kept small (equivalent to approximately 0.05 mm of fiber segment) to improve the resolution of the myosin heavy chain bands (types I, IIa, IIx and IIb). Electrophoresis was performed at 120 V for 24 h with a Tris–glycine electrode buffer (pH 8.3) at 15°C (SE 600 vertical slab gel unit, Hoefer Scientific Instruments). The gels were silver-stained and subsequently scanned in a soft laser densitometer (Molecular Dynamics) with a high spatial resolution (50 μm pixel spacing) and 4096 optical density levels.

**Table 3:** Energy of non-bonded and electrostatic interactions in the actomyosin complex in the presence or absence of the Asp286Gly mutation.

		With Asp286		With Asp286Gly	
	Residue	Non-bonded	Electrostatic	Non-bonded	Electrostatic
**Actin**	Cys285	−29.21	8.08	−26.79	39.56
	Asp286	−28.97	9.13	−14.56	40.70
	Ile287	−19.58	−0.59	−30.84	10.21
	Asp288	−22.60	17.02	−34.56	19.14
	Arg289	−29.44	−1.74	−29.89	−1.12
**Actin neighbor**	Arg62	−45.71	−206.14	−44.64	−205.10
	Gly63	−15.79	64.37	−14.21	64.16

The energy unit is kJ.mol^−1^. Energy computations were done in vacuo with the GROMOS96 implementation of Swiss PDB Viewer and without reaction field.

**Figure 4:**
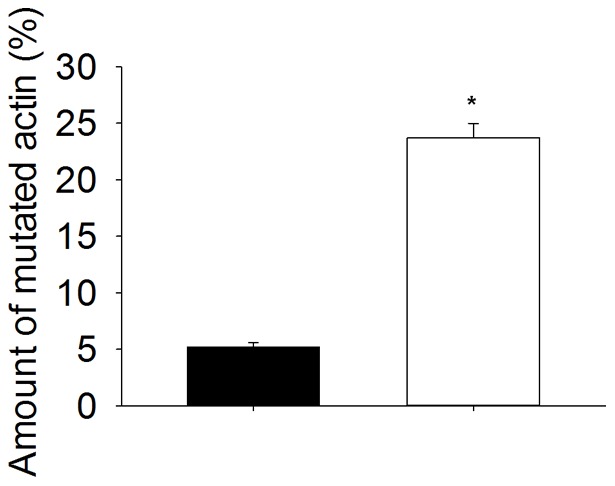
Proportion of mutated actin in Tg(*ACTA1*)^Asp286Gly^ mice. Data from various muscles (▪, slow- vs. □, fast-twitch muscles) are presented as means (SEMs). Stars indicate significant differences between slow- and fast-twitch muscles (p<0.05).

MyHC and actin relative contents in fibers were determined by 12% SDS-PAGE. The acrylamide concentration was 4% (wt/vol) in the stacking gel and 12% in the running gel, and the gel matrix included 10% glycerol. The gels were stained with Coomassie blue (0.5 g brilliant blue, 225 ml methanol, 225 ml distilled H_2_0, and 50 ml acetic acid). The relative contents were then calculated from the densitometric scanning (please see above).

**Figure 5:**
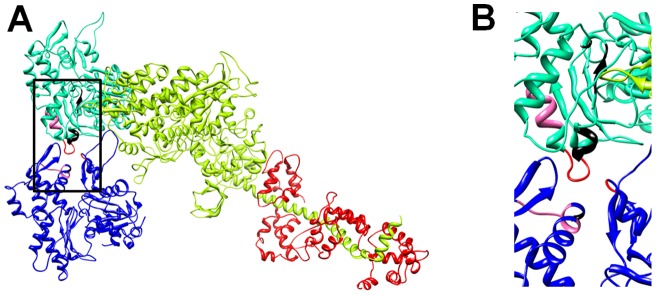
Myosin cross-bridge in presence of Asp286Gly, Glu205Asp and Asp292Val. General [A] and particular view [B] of the modified regions. The model was originally designed by Lorenz and Holmes [17]. Changes related to Asp286Gly appear in red, those associated to Asp292Val in black and the ones linked to Glu205Asp in pink.

### Actomyosin modeling

The model used in the present study has extensively been described elsewhere [17]. It was visualized with UCSF Chimera [18] and Swiss PDB viewer. Energy computations in the absence or presence of the actin mutation were done *in vacuo* with the GROMOS96 implementation of Swiss PDB viewer.

### Mass spectrometry

This was performed as outlined in [9] using samples coming from various slow- and fast-twitch muscles (gastrocnemius, diaphragm and soleus).

### Statistical analyses

Because of a MyHC type IIb fiber predominance in EDL muscles of wild-type and Tg(*ACTA1*)^Asp286Gly^ mice, the comparison was restricted to cells expressing this MyHC isoform. Data are presented as means ± standard error of the means (SEMs). Sigma Stat software (Jandel Scientific) was used to generate descriptive statistics. The unpaired Student’s t-test was applied, and in cases where the data did not meet the criteria of normality (Kolmogorov-Smirnov test, p<0.05), the non-parametric Mann-Whitney rank-sum test was performed. Otherwise, regressions were performed and relationships were considered significantly different from zero at p<0.05.

## Results

### Single muscle fiber mechanics

Steady-state isometric force at saturating [Ca2^+^] (pCa 4.50), was significantly lower in fibers from Tg(*ACTA1*)^Asp286Gly^ mice when compared with wild-type animals (Table 2). To gain insights into the underlying mechanisms, we performed stiffness measurements. Thus, step changes in fiber length were imposed (releases of 0.15, 0.3 and 0.5% of fiber length and stretches of the same amplitudes). Stiffness was defined as the slope of the linear regression of the relationship between the peak force response and the length change (Fig. 1). As for force, active stiffness (S_a_) at saturating [Ca^2^+] (pCa 4.50) was significantly reduced in fibers from Tg(*ACTA1*)^Asp286Gly^ rodents when compared with wild-type mice (Table 2). On the other hand, under rigor conditions, where all myosin heads are attached due to a very slow dissociation rate [19], rigor stiffness (S_r_) was not significantly affected by the Asp286Gly actin mutation (Table 2). These active and rigor stiffness data allowed the calculation of the fraction of strongly bound myosin cross-bridges (*f*_xb_) using the following equation: *f*_xb_  =  (S_a_/S_r_)/(2-(S_a_/S_r_) [20]. *f*_xb_ was significantly smaller in fibers from Tg(*ACTA1*)^Asp286Gly^ animals when compared with wild-type mice (Table 2).

Besides the slope, the x-axis intercept of the relationship between the peak force response and the length change was measured (y_0_) at various [Ca^2+^] (pCa between 6.30 and 4.50). The y_0_-steady-state isometric force relationship was then constructed for each fiber and found linear (Fig. 1). The y-axis intercept gave an index of the strain of individual myosin heads attached to actin (y_0_*) whereas the slope estimated the compliance due to the structures in series with the actomyosin interactions (C_f_) as well as the extra-compliance due to the attachment of the fiber ends to the force transducer and lever arm (C_e_) [21], [22]. C_f_ + C_e_ as well as y_0_* were not significantly different between fibers from Tg(*ACTA1*)^Asp286Gly^ and wild-type mice (Table 2).

In addition to all the above measurements, the rate constant of force redevelopment (*k*_tr_) was evaluated. Once steady-state isometric force was reached at saturating [Ca^2+^] (pCa 4.50), a slackening of 20% of the original fiber length was introduced within 1–2 ms at one end of the fiber, resulting in a rapid reduction of force to near zero. This was followed by a brief period of unloaded shortening (20 ms), after which the preparation was quickly restretched to its original length and the force recovered to its original steady-state value. As described previously [23], *k*_tr_ was estimated by a linear transformation of the half-time of force redevelopment (t_1/2_) as follows [24]: *k*_tr_  = 0.693/t_1/2_. *k*_tr_ was not altered by the Asp286Gly actin mutation (Table 2).

Maximum unloaded shortening velocity (V_0_) was also calculated. Once steady-state isometric force was reached at saturating [Ca^2+^] (pCa 4.50), nine slacks of various amplitudes were rapidly introduced (within 1–2 ms) at one end of the fiber [25]. Slacks were applied at different amplitudes ranging from 7 to 13% of the fiber length [15], . The fiber was re-extended between releases while relaxed in order to minimize changes in sarcomere length. During the slack test, the time required to take up the imposed release was measured from the onset of the length step to the beginning of the tension redevelopment. A straight line including four or more data points was fitted to a plot of release length versus time, using least-squares regression. The slope of the line divided by the fiber segment length was recorded as the maximum unloaded shortening velocity (V_0_) for that fiber segment [25]. V_0_ was enhanced in fibers from Tg(*ACTA1*)^Asp286Gly^ rodents when compared with wild-type mice (Table 2).

### Actin expression

Even though some sarcomeric areas appear disrupted [9], the relative content of actin was unaffected by Asp286Gly as shown by the unchanged myosin to actin ratio (Fig. 2).

### Actomyosin modeling

A model of strong myosin head binding to actin in the rigor-like state was used [17]. In this model, replacing aspartic acid by glycine at position 286 altered the energy of some non-bonded interactions. Residues of the mutated actin monomer (Cys285, Asp286Gly, Ile287, Asp288 and Arg289) as well as of neighboring actin monomers (Arg62 and Gly63) were affected (Table 3 and Fig. 3). However, no difference was seen for myosin peptides. Thus, the change in the actin-actin interface may disturb myosin binding differently than altering the energy of interactions at the actomyosin interface.

## Discussion

The reduced steady-state isometric force at the muscle fiber level and the overall skeletal muscle weakness in Tg(*ACTA1*)^Asp286Gly^ mice may not primarily be due to the presence of non-contractile areas (nemaline bodies, myofibrillar disruption and protein aggregates) but rather to the effect of the “poison-protein” Asp286Gly on actin-myosin interactions.

In another transgenic mouse line also expressing the Asp286Gly variant but fused with the reporter molecule enhanced green fluorescent protein (EGFP; Tg(*ACTA1*)^Asp286Gly-EGFP^) [10], the amount of Asp286Gly has been shown to vary between muscles and ages [10]. Since the constructs employ the same promoter and enhancer than the Tg(*ACTA1*)^Asp286Gly^ mice, a similar variation among muscles and ages can be extrapolated [9], [10]. Considering this, in the present study, we focused on fibers from a fast-twitch muscle (MyHC IIb from EDL) where the highest level of mutant actin monomers is expressed (Fig. 4) and we observed a dramatic reduction in the steady-state isometric force production. This depression can arise from either a reduced force-generating capacity per individual actomyosin interaction and/or a lower number of strongly bound myosin cross-bridges during maximal activation. The strain of individual myosin heads attached to actin (y_0_*) was not affected by Asp286Gly (Table 2). This is in line with two previous *ACTA1*-related mutations, i.e., Asp292Val located in the actin-actin interface and Pro332Ser positioned close to the nucleotide binding site [26], but differs from three other defects where actomyosin force is enhanced, i.e., Met132Val and Lys336Glu also located near the nucleotide attachment region [26] and Phe352Ser positioned at the actomyosin interface [27]. Therefore, here, the reduced steady-state isometric force has to originate from a smaller number of myosin cross-bridges strongly bound to actin monomers as attested by stiffness data (Table 2). Such a deficit is unlikely to be directly related to the presence of non-contractile areas in the skeletal muscles of Tg(*ACTA1*)^Asp286Gly^ mice. Indeed, rigor stiffness where all myosin molecules are attached was not modified (Table 2).

Taking into account that the fraction of strongly bound myosin cross-bridges calculated in the present study (*f*_xb_) is proportional to f_app_ / (f_app_ + g_app_) and that *k*_tr_ is known to depend on f_app_ + g_app_ and V_0_ has g_app_ as rate-limiting step [23], [28], with f_app_ being the rate constant for attachment and g_app_ the rate constant for detachment [29], one may suggest that the decrease in the steady-state isometric force production with Asp286Gly is a direct consequence of altered myosin head binding to actin monomers, more precisely of a modification of the time spent by individual myosin molecules in a strongly attached force-producing conformation (a slower f_app_ together with a faster g_app_). This phenomenon has already been observed for various congenital myopathy-related mutations altering thin filament proteins such as tropomyosin [14], [15], [16], [30] and nebulin [31].

Besides a change in cross-bridge kinetics to explain the alteration of myosin binding to actin with Asp286Gly, one may hypothesize an inefficient thin filament movement. Indeed, the process of myosin head attachment to actin is controlled by the activation of thin filament proteins including tropomyosin. Such activation is triggered by Ca^2+^ as well as by myosin cross-bridges [32]. According to the steric blocking model [32], [33], in the absence of Ca^2+^, the position of tropomyosin on the thin filament essentially blocks the attachment of myosin heads to actin. When Ca^2+^ is added, troponin undergoes conformational changes, allowing tropomyosin to move on the thin filament, partially exposing myosin-binding sites on actin. Full tropomyosin activation and complete exposure of myosin binding sites on actin happens when myosin cross-bridges further displace tropomyosin. In the present study, as rigor stiffness was maintained (Table 2) and as the Asp286Gly actin mutation is located far away from tropomyosin binding sites, i.e., Asp25, Arg28, Arg147, Lys326, Lys327, Lys328, Pro333 and Glu334 [34], an inefficient tropomyosin movement may not occur.

How would Asp286Gly affect myosin cross-bridge kinetics? This is still unclear. However, according to the energy computations (Table 3), the mutation changes the stability of various amino acids of the protein and of the adjacent actin molecules. Thus, Asp286Gly modifies the strength of the bonds between numerous residues (Table 3) and therefore, it is likely to affect the actin-actin interface (Fig. 3). A few other mutations leading to congenital myopathies and skeletal muscle weakness are positioned in the same region, e.g., His40Tyr, Ile64Asn, Glu205Asp, Gln263Leu, Gly268Cys and Asp292Val [26]. Out of these six defects, only two have been partially functionally characterized. As mentioned previously, Asp292Val does not affect the force-generating capacity per individual actomyosin interaction but slightly decreases the sliding speed of actin over myosin molecules. This is in contrast with Glu205Asp where the actin motility speed is increased [26]. To better understand how Glu205Asp and Asp292Val alter the actin-actin interface and diverge from Asp286Gly, we performed further molecular energy computations in the presence or absence of the mutants (Fig. 5). As expected, Asp286Gly, Asp292Val and Glu205Asp all modify the adjacent actin molecules but with some mutation-specific differences. This is supposedly due to the various F-actin contact locations having discrete functional implications [35]. Hence, *ACTA1* mutations located in the F-actin contact region have various dysfunctions and no common cascades of molecular events can be found. Restoring the actin-actin interface would, nevertheless, constitute one potential therapeutic intervention in the future. Another possibility would be to directly modulate myosin cross-bridge formation and reverse the decreased steady-state isometric force at the muscle fiber level. Existing pharmacological drugs, for cardiac muscle, increase contractility in such a manner through various cascades [36], [37], [38] and may thus be applicable to some patients with *ACTA1* mutations.

In conclusion, taken together, the present data suggest that the Asp286Gly *ACTA1* mutation modifies the actin-actin interface and triggers numerous functional alterations. It notably prevents proper myosin binding to actin monomers, limiting the amount of strongly bound cross-bridges. At the cell level, this dramatically decreases the steady-state isometric force production and overall significantly contributes to skeletal muscle weakness in Tg(*ACTA1*)^Asp286Gly^ mice. All these phenomena have to be considered when designing future drug therapies.
